# Bioactive Compounds, Nutritional Quality and Antioxidant Capacity of the Red-Fleshed Kirkwood Navel and Ruby Valencia Oranges

**DOI:** 10.3390/antiox11101905

**Published:** 2022-09-26

**Authors:** Jaime Zacarías-García, Laura Pérez-Través, José-Vicente Gil, María-Jesús Rodrigo, Lorenzo Zacarías

**Affiliations:** 1 Instituto de Agroquímica y Tecnología de Alimentos (IATA), Consejo Superior de Investigaciones Científicas (CSIC), 46980 Valencia, Spain; 2 Food Technology Area, Faculty of Pharmacy, University of Valencia, 46100 Valencia, Spain

**Keywords:** red-fleshed oranges, maturation, nutritional quality, bioactive compounds, antioxidant

## Abstract

Kirkwood Navel and Ruby Valencia are two spontaneous bud-mutations of the ordinary Washington Navel and Valencia late oranges characterized by the red coloration of their flesh. The purpose of this study was to analyze the physiological features, internal fruit quality, contents of relevant bioactive compounds and antioxidant capacity in the pulps of the red-fleshed fruits compared with the ordinary oranges during late development and maturation. In general, the content of sugars, organic acids, vitamin C, tocopherols, total phenolics and flavonoids, the hydrophilic antioxidant capacity and their changes during maturation were similar in the red-fleshed oranges and in the corresponding blond oranges. However, the mature Ruby fruits contained lower concentrations of sugars, malic and succinic acid and higher levels of citric acid than the ordinary Valencia. The major difference between the pulps of the Kirkwood and Ruby oranges and those of the ordinary oranges was the higher lipophilic antioxidant capacity and SOAC (singlet oxygen absorption capacity) of the former. Together, the high and unique content and composition of carotenoids in Kirkwood and Ruby may contribute to an enhanced antioxidant capacity without any detrimental effects on other fruit-quality attributes, making these varieties good sources of phytochemicals for the fresh-fruit and juice-processing citrus industries.

## 1. Introduction

*Citrus* is one of the most important commercial fruit crops worldwide in terms of production, market trade and consumption. The demand for and acceptance of citrus fruits for fresh consumption or juice production is highly variable across different national and international markets, depending on the consumer choices and preferences but, in general, it is mainly based on the external appearance, organoleptic properties, nutritional quality and health-related benefits [1].

The consumption of citrus fruits is associated with anti-inflammatory effects, obesity control, a decrease in the incidence of cardiovascular diseases and a reduction in the risk of certain cancers [2,3,4]. Therefore, the current concern over the prevention of health problems through nutrition is leading to intensive studies of the nutritional composition and antioxidant properties of citrus fruits. In this sense, oxidative stress plays a pivotal role in the development of human diseases and evidence indicates that the several beneficial effects of citrus metabolites in human health are associated with their antioxidant activity [5,6].

The content and composition of nutrients and bioactive and antioxidant metabolites in citrus fruits vary widely among the different species and varieties, tissues and maturation stages, influencing their quality, nutritional value and health-promoting effects [7]. Orange fruits (*Citrus sinensis*) are relevant sources of various nutrients and phytochemicals, including carbohydrates, protein, lipids, vitamins, mineral elements, phenolics, limonoids and carotenoids among others [4,7,8]. Sugars are the main nutrients of the fruits of most citrus species and, with organic acids, have a strong impact on the characteristic flavour of the fruit, playing a crucial role in its organoleptic properties and consumer acceptance [1,3]. Carotenoids are fat-soluble compounds responsible for the coloration of the peels and pulps of the fruits of most citrus species and varieties, and their content is a key issue in their commercial acceptability [9]. The diversity of pigmentation in citrus fruits is mainly due to the genetic and environmental variability in the content and composition of carotenoids [10]. Citrus phenolics have been widely characterized in orange fruits, and phenolic acids and flavonoids are the largest group of naturally occurring phenolic compounds; they are present as both free state (aglycones) and glycosyl derivatives [4]. The flavonoid composition in fruits of different citrus species has been studied in depth; it has been shown that their content may fluctuate during fruit development and maturation [11,12]. Flavonoids, in general, display an ample range of biological and health-related activities, such as their anti-inflammatory and protective role against chronic diseases, including cardiovascular diseases and diabetes [12,13].

Citrus fruits are also important sources of vitamins [7,8]. For example, citrus fruits are recognized worldwide as some of the main sources of vitamin C (ascorbic acid). Ascorbic acid is the most abundant vitamin in orange fruits and, as many authors suggest, the major antioxidant in *Citrus* [13,14]. Although the accumulation of L-ascorbic acid in the pulp of citrus fruits is, in general, moderate in comparison with other fruits, such as kiwi or strawberry, the large consumption of fresh oranges and orange juice made from this crop make it a major source of vitamin C in human nutrition [15]. On the other hand, tocopherols are plant isoprenoids belonging to the chemical family of tocochromanols. With the exception of tocotrienols, they are the only natural compounds that exhibit vitamin E activity in animal cells, with α-tocopherol being the most potent vitamin E form [16,17]. The target and synergistic activity of these phytochemicals and others determine the antioxidant capacity of citrus fruits and, therefore, most of their health-related properties [6]. Several recent reviews gather the latest insights into the biological effects of complex mixtures of phytochemicals from food matrices [18,19,20]. More specifically, several studies proposed different mechanisms of action or interactions between plant antioxidants. For instance, it has been demonstrated that vitamin C is able to regenerate oxidized vitamin E and carotenoids [21,22]. Additional evidence suggested that the combination of flavonoids (rutin) and carotenoids (lycopene and lutein) leads to synergistic effects that prevent low-density lipoprotein oxidation [23].

The accumulation of the carotene lycopene in the fruit pulp confers a particular bright-reddish coloration, but this is an unusual feature in the genus *Citrus*, restricted to a few varieties and mutants [9]. The red-fleshed Hong Anliu and Cara oranges, identified in China and Venezuela, respectively, have been intensively investigated during last decade. Several studies focused on the analysis of their carotenoid composition [24,25,26,27], accumulation of vitamins and bioactive compounds [28,29], antioxidant activity and cytoprotective effects [30,31,32] and alterations in other metabolic pathways related to fruit quality in comparison with standard varieties [33,34,35,36,37].

The availability of new red-fleshed orange varieties is of special relevance to the provision of novel products with distinctive value for local and international markets, but also to understand the biochemical basis of lycopene accumulation in the pulps of citrus fruits and whether this feature may alter other parameters of fruit quality. We recently reported the characterization of carotenoid metabolism during development and maturation in two new red-fleshed orange mutants: Kirkwood Navel (referred in this work as Kirkwood or K) and Ruby Valencia (Ruby or R). These spontaneous bud-mutations are derived from the ordinary Navel and Valencia late oranges, respectively, and are already being commercially propagated in order to check their adaptation to Mediterranean growing and environmental conditions [38]. The pulps of both mutants contain higher amounts of total carotenoids than the ordinary Navel and Valencia oranges from the early stages of fruit development and, moreover, accumulate large concentrations of phytoene and phytofluene, moderate amounts of lycopene, low levels of β-carotene and minor concentrations of violaxanthin compared to the ordinary varieties [38]. Hence, the appealing coloration and the high concentration of carotenes make these new orange varieties rich sources of carotenoids for consumption and novel food products with potential benefits for health.

The objective of this work was to conduct a comparative analysis of the chemical composition, nutritional quality and antioxidant capacity in the fruits of the red-fleshed varieties, Kirkwood and Ruby, and the corresponding standard varieties, Navel and Valencia, respectively, in order to provide reliable data on their fruit-quality characteristics under edaphoclimatic Mediterranean conditions. We report, for the first time, the evolution of internal quality-related parameters, such as sugars and organic acids, from the late stages of fruit development to full maturation, as well as the accumulation of vitamins and other bioactive compounds, such as ascorbic acid, tocopherols, total phenolics and flavonoids. Furthermore, the relevance of these changes is complemented by the analysis of the carotenoid composition in the fruit pulp at two maturity stages and the assessment of the antioxidant capacity by different in vitro assays.

## 2. Materials and Methods

### 2.1. Plant Material

**Figure 1:**
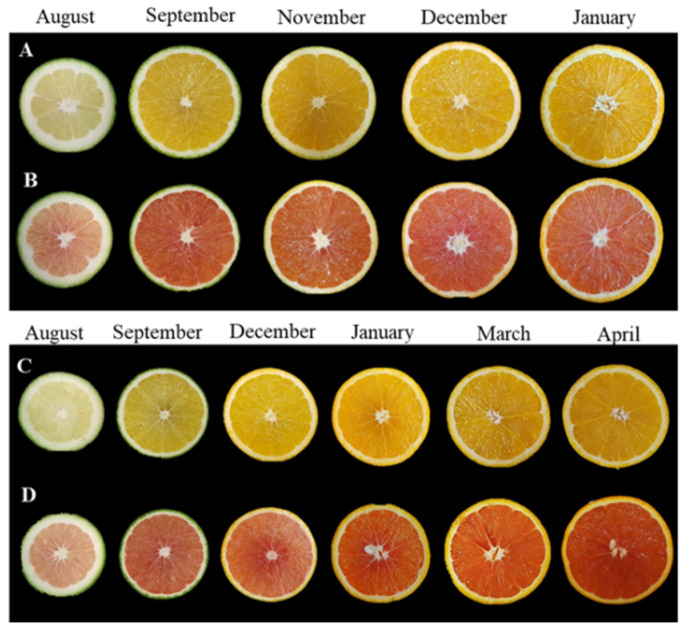
Internal appearance of the Navel (**A**), Kirkwood (**B**), Valencia (**C**) and Ruby (**D**) orange fruits used in this study.

Fruits of the two ordinary sweet orange (*Citrus sinensis*) genotypes, Washington Navel (cv. Foios, N) and Valencia late (cv. Midknight, V) and those of the red-fleshed varieties, K and R, were cultivated at the Fundación ANECOOP (Museros, Valencia, Spain). Washington Navel oranges are characterized by precocious maturity and bear large seedless fruits. They are the main orange group produced for fresh consumption. On the other hand, Valencia late oranges belong to the Blancas botanical group; they are characterized by the absence of navel in their fruits, late maturity and the absence of a bitter flavour in their juices [39]. Fruits of each genotype were harvested from adult trees grafted under Citrange Carrizo rootstock (*Citrus sinensis* × *Poncirus trifoliata*), grown in the same orchard and under the same agronomical and environmental conditions [14]. N and K fruits were harvested at five stages during the following months: August (immature green), October (green–breaker), November (breaker), December (mature) and January (fully mature) and those of V and R were harvested at six stages: August (immature green), October (mature green), December (green–breaker January (breaker), March (mature) and April (fully mature) (Figure 1).

At each selected stage, at least 50 fruits per variety were harvested and delivered to the laboratory. Fruits were picked up from the external canopy; they were of similar size and free of external defects. Color of the flavedo and pulp was measured and small pieces (around 1 cm^3^) of the pulp containing juice vesicles without segment membranes were excised and frozen in liquid nitrogen, ground to fine powder and stored at −80 °C until analysis. Juice was extracted with a domestic electric hand squeezer (Citromatic MPZ22, Braun, Barcelona, Spain), filtered through a metal filter (0.8 mm pore size), frozen in liquid nitrogen and stored at −20 °C until analysis. Fruit samples were harvested during two consecutive seasons (2018–2019; 2019–2020) and data reported here are representative of one season unless otherwise indicated.

### 2.2. Fruit Weight, Size, Color Index and Internal Maturity Determination

At least 20 fruits were individually weighed and height and diameter were measured for each variety and harvest date. Color of the flavedo and pulp was measured using a CR-400 Minolta chromameter on three different positions and is expressed as the a/b Hunter ratio. Color measurements were not determined in fruits harvested in August since the pulp diameter was too small to obtain a reliable determination in three different positions. Data of color index for each cultivar are the means ± SD of at least 10 fruits.

Total soluble solids (TSS) and total titratable acidity (TA) of the juice were determined using a digital refractometer PAL-BX/ACID1 (ATAGO, Tokyo, Japan). TSS is expressed as °Brix and TA as mg citric acid/100 mL of juice; maturity index (MI) was calculated as the TSS/TA ratio. Determination of internal maturity was analyzed in fruits of the four varieties at the different harvest times, except for those harvested in August.

### 2.3. Sugar and Organic Acid Determination

Sugars and organic acids were determined essentially as described by Pérez-Través et al. [40]. Pulp tissue (2 g) was homogenized with 2 mL of Milli-Q water and then centrifuged for 10 min at 3000 g at 4 °C. The supernatants were diluted 3-fold and filtered through a 0.22-micrometer nylon filter (Symta, Madrid, Spain). A liquid chromatograph (Thermo Fisher Scientific, Waltham, MA, USA) equipped with a refraction-index detector was used. For glucose, fructose, citric acid, malic acid, quinic acid and succinic acid, a HyperREZTM XP Carbohydrate H+ 8-micrometer column (Thermo Fisher Scientific) was used, protected by a HyperREZTM XP Carbohydrate (Thermo Fisher Scientific) guard column. The conditions used in the analysis were as follows: eluent, H_2_SO_4_ 1.5 mM; flux, 0.6 mL/min; and oven temperature, 50 °C. For sucrose determination, a Pb column was used (Hi-Plex Pb, 300 × 7.7 mm, Agilent Technologies (Santa Clara, CA, USA); the conditions used in this analysis were: eluent, Milli-Q water; flux, 0.6 mL/min; and the column temperature was set at 50 °C. Samples were extracted twice and results are the mean of two replicates (mean ± SD).

### 2.4. L-Ascorbic-Acid Determination

L-Ascorbic acid from the pulp of the fruit was extracted and determined essentially as described in Alòs et al. [41], using a Waters ACQ Arc SysCore HPLC equipped with a DAD, Empower 3 software and an Ultrabase C18 column.

### 2.5. Tocopherol Determination

Tocopherols in the pulp were extracted and quantified essentially as described in Rey et al. [16], using a Waters HPLC system (Acquity^®^ Arc™, Waters, Barcelona, Spain) coupled with a fluorescence detector (2475 FLR Detector, Waters, Barcelona, Spain) and a YMC C30 column (150 × 4.6 mm, 3 μm) (Teknokroma, Barcelona, Spain). Identification and quantification of the different tocopherols was achieved by comparison with the retention times and peak areas of authentic standards of δ-, γ- and α-tocopherol (Sigma-Aldrich, Barcelona, Spain), and the concentrations expressed as μg/g of fresh weight. Samples were extracted twice and results are the mean of two replicates (mean ± SD).

### 2.6. Carotenoid Determination

Carotenoids were extracted and analyzed in the pulps of mature fruits of the four varieties (December and January for Navel oranges, and March and April for Valencia oranges) as described by Rodrigo et al. [26], using a Waters a liquid chromatography system (HPLC) equipped with a 600E pump, a photodiode array detector (DAD), model 2998 and Empower3 software (Waters, Barcelona, Spain). A C30 carotenoid column (250 × 4.6 mm, 5 μm) was coupled to a C30 guard column (20 × 4.0 mm, 5 μm) (YMC, Teknokroma, Spain). The carotenoids were identified by absorbance spectra and retention time; peaks were integrated at their individual maximal wavelength and their contents were calculated using the appropriate calibration curves of lycopene (Extrasynthese) for lycopene, neurosporene and δ-carotene, lutein (Sigma), β-carotene (Sigma), β-cryptoxanthin (Extrasynthese), zeaxanthin (Extrasynthese), anteraxanthin (CaroteNature) for anteraxanthin and mutatoxanthin and violaxanthin (CaroteNature) for violaxanthin isomers and luteoxanthin. Phytoene, phytofluene and ζ-carotene were previously purified by thin-layer chromatography from carotenoid extracts of Pinalate orange fruits [26]. The spectroscopic characteristics of all carotenoids detected in the pulps of Navel, Kirkwood, Valencia and Ruby oranges are shown in Table S1.

### 2.7. Analysis of Total Phenolics and Flavonoids

Phenolic content was determined by the method of Folin–Ciocalteu, as described by Singleton and Rossi [42], with slight modifications. Briefly, 0.5 g of pulp tissue were extracted with 5 mL of MeOH using a homogenizer (Polytron, Eschbach, Germany). The extract was centrifuged for 10 min at 3000 g at 4 °C and the supernatant used to estimate the phenolic content. A 50-microliter aliquot of the extract was mixed with 500 μL of solution (Na_2_CO_3_ 2% and NaOH 0.1 M) and incubated for 15 min at room temperature in darkness. Next, 50 μL of Folin–Ciocalteu reagent were added (diluted 50% with purified water) and incubated for 30 min. The absorbance was measured at 724 nm in a UV/Vis microplate spectrophotometer (Multiskan FC, Thermo Scientific, Waltham, Massachusetts, USA) and compared with a standard curve of gallic acid (Sigma-Aldrich, Barcelona, Spain). Results were expressed as mg of gallic-acid equivalents (GAE) per 100 g of fresh weight. Determinations were performed per triplicate in each extract.

Total flavonoids were determined in the same supernatant used to analyze total phenolics following the method described by Lafuente et al. [43]. Two supernatant aliquots of 100 μL were diluted with 400 μL of Milli-Q water and 30 μL of 5% NaNO_2_. After 5 min of incubation at room temperature, 30 μL of 10% AlCl_3_ were added and the reaction was stopped by adding 200 μL of 1 N NaOH. Flavonoid contents were determined by UV/Vis microplate spectrophotometer comparing the absorbance at 350 nm with a standard curve of hesperidin (Sigma-Aldrich, Barcelona, Spain). Results were expressed as mg of hesperidin equivalents (HesE) per 100 g of fresh weight. Determinations were performed per triplicate in each extract.

### 2.8. Flavonoid Determination

Flavonoids were extracted and purified from pulp tissue as follows. Briefly, pulp tissue (0.5 g) was extracted with 5 mL of Milli-Q water centrifuged at 3000 g for 30 min at 4 °C. Next, the extract was subsequently filtered through a 0.45-micrometer PVDF filter (13 mm diameter, Análisis Vínicos, Tomelloso, Ciudad Real, Spain). Flavonoid content was determined by HPLC-DAD. Separation of flavonoids was carried out with a XBridgeTM BEH C18 (4.6 × 150-millimeter, 2.-micrometer Column XP), maintained at 40 °C, and a secondary gradient elution with water 0.1% TFA (solvent A) and acetonitrile 0.1% TFA (solvent B) at a flow rate of 1 mL/min as follows. From 0 to 5 min 100% A, 5 to 30 min 95% A and 5% B, 30 to 33 min 50% A and 50% B and from 33 to 37 min 100% B. Wavelength was registered from 220 to 550 nm and flavonoids were quantified at 280 nm. Identification and quantification of the different flavonoids was achieved by comparison with the retention times and peak areas of authentic standards of hesperidin, narirutin, naringin, eriocitrin, dydimin and rutin (Sigma-Aldrich, Barcelona, Spain). Concentrations are expressed as mg/100 g of fresh weight. Samples were extracted twice and the results are the mean of two replicates (mean ± SD).

### 2.9. Determination of the Antioxidant Capacity

The hydrophilic antioxidant capacity (HAC) was determined by the DPPH free-radical assay (2,2-diphenyl-1-picrylhydrazyl), as described by Rey et al. [44]. The assay was replicated twice and a curve of ascorbic acid was used as a standard. Results were expressed as mg of ascorbic acid equivalents (AsAE) per 100 g of fresh weight. DPPH-scavenging capacity was expressed as inhibition percentages by Formula (1):

% DPPH scavenging capacity = [(517 nmAcontrol − 517 nmAsample)/517 nmAcontrol] × 100 (1)

The HAC was also determined in the same supernatant used in DPPH assay by using FRAP (ferric reducing antioxidant power) assay, as described by Benzie and Strain [45], with some modifications. First, a reagent was prepared with 10 mM TPTZ (2,4,6-tripyridy-Striazine, Sigma), 20 mM FeCl_3_.6H_2_O and 300 mM acetate buffer, pH 3.6, at 1:1:10, *v*:*v*:*v* and incubated in a bath at 37 °C for 10 min. In a 96-well plate 10 μL of each sample were mixed with 290 μL of FRAP reagent and incubated at 37 °C in darkness for 30 min. Thereafter, the absorbance change of the mixture was determined in a UV/Vis microplate spectrophotometer (Multiskan FC, Thermo Scientific, Madrid, Spain) at 595 nm. The assay was replicated twice and a curve of ascorbic acid was used as a standard. Results were expressed as mg of ascorbic acid equivalents (AsAE) per 100 g of fresh weight.

The ABTS (2,2′-azino-di-(3-ethylbenzthiazoline sulfonate) assay was used to determine antioxidant capacity in comparison to a standard antioxidant, Trolox (6-hydroxy-2,5,7,8-tetramethylchroman-2-carboxylic acid) (Sigma, Madrid, Spain). Total antioxidant capacity was quantified as described by Legua et al. [46], with slight modifications, which enabled us to determine antioxidant capacity due to both hydrophilic and lipophilic compounds in the same extraction. Absorbance of the extracts was determined with a UV/Vis microplate spectrophotometer (SPECTROstar^®^ Omega, BMG Labtech, Offenburg, Germany) at 730 nm. A calibration curve was drawn with Trolox (Sigma, Madrid, Spain) and results are expressed as Trolox equivalent antioxidant capacity (TEAC) per 100 g of fresh weight.

### 2.10. Analysis of Singlet Oxygen Absorption Capacity (SOAC)

Analysis of SOAC in the pulp of the fruits was performed as previously described by Rey et al. [44]. The capacity of the extract to scavenge the **^1^**O**_2_** radical was determined using endoperoxide (EP, Invitrotech, Kyoto, Japan) as a singlet oxygen generator and 2,5-diphenyl-3,4-benzofuran (DPBF, Sigma–Aldrich, Barcelona, Spain) as an UV-Vis absorption probe in a 96-well quartz glass microplate (Hellma, Müllheim, Germany). Changes in absorbance at 413 nm were monitored for 90 min at 35 °C using a UV-Vis spectrophotometer microplate reader; α-Tocopherol (Sigma–Aldrich, Barcelona, Spain) was used as a blank standard. The SOAC value was calculated by Formula (2):

(t1/2 sample − t1/2 blank)/(t1/2 α-toc − t1/2 blank) × ([α-toc, g L^−1^]/[sample, g L^−1^]) (2)

Each sample was analyzed in two independent assays and samples were run in triplicate in each plate.

### 2.11. Statistical and Principal Component Analysis (PCA)

Results are presented as the mean of two biological replicates ± standard deviation (SD). Differences between the red-fleshed and the corresponding ordinary variety for each harvest date were analyzed by *t*-test (*p* < 0.05) using XLSTAT software 2019.3.2 (Addinsoft, Paris, France). The correlations between bioactive compounds and antioxidant capacity measured by different assays were examined by Pearson’s correlation (*p* < 0.05). Two kinds of principal component analysis (PCA) were built. The first PCA was set up using data for vitamin C, tocopherols, total phenolics, total flavonoids, DPPH, FRAP, ABTS-H, ABTS-L by XLSTAT software. The second PCA was built with the same variables and levels of carotenoids. The data for total carotenoids for this second PCA analysis were obtained from our previous work [38], using the same biological samples.

## 3. Results and Discussion

The red-fleshed oranges, Kirkwood Navel (K) (https://www.biogold-em.com/assets/va---spec-sheet---kirkwood-red-navel-(print).pdf) (accessed on 19 September 2022) and Ruby Valencia (R) (https://www.biogold-em.com/assets/va---spec-sheet---ruby-valencia-(print).pdf) (accessed on 19 September 2022), originated from spontaneous bud mutations of the blond-colored Palmer Navel and Olinda Valencia oranges, respectively, grown in commercial orchards. These varieties were developed under agronomical and environmental Mediterranean conditions, maintaining the distinctive color singularities and carotenoid complements in the pulp [34]. These mutants are characterized by their accumulation of large concentrations of the colorless carotenes, phytoene and phytofluene, considerable amounts of lycopene, low levels of β-carotene and reduced amounts of violaxanthin, the major carotenoid in orange fruits, compared to their ordinary varieties N and V, respectively. In the current study, we report a comprehensive analysis of the content of relevant fruit-quality components and the antioxidant capacity of these new red-fleshed varieties in comparison to the corresponding ordinary oranges.

### 3.1. Physiological Parameters, Color and Internal Quality in the Red-Fleshed Kirkwood Navel and Ruby Valencia, and the Ordinary Navel and Valencia Oranges

The fruits’ growth, measured as changes in diameter and height, increased markedly from August to October in the four genotypes, corresponding to the cell-enlargement period (stage II) of citrus-fruit development, in which the increase in pulp size is responsible for most of the growth [47]. From October to January, for N and K, and from October to April, for V and R, the rate of fruit growth was reduced and most of the maturation-related events occurred during this period (stage III). During this period, no differences in fruit size were detected between the two red-fleshed genotypes and the corresponding ordinary oranges (Table 1 and Table 2). These results indicate that final fruit size at harvest is similar for each mutant with respect to its standard variety, which is line with observations in other red-fleshed oranges [25,27].

The main feature of K and R mutants is the inner coloration of the fruit. The internal appearance of the Navel, Kirkwood, Valencia and Ruby orange fruits is shown in Figure 1. The a/b Hunter ratio revealed that the colors of the pulps of the K and R fruits were completely different compared to their respective standard lines (Table 1 and Table 2). In the fruits of the N and V, the a/b values were negative and slightly positive, corresponding to green–yellow shades, but in both mutants, the color of pulp showed considerably higher positive values, indicating a red coloration of the pulp. These differences in color parameters were patent from the early stages of development, indicating that the alteration in pulp pigmentation was not linked to the maturation process, as has been previously suggested [38]. Moreover, in the fruits of both mutants, the a/b values decreased during maturation; this was probably related to dilution caused by the expansion of the pulp (Table 1 and Table 2). The color index of the peel changed from negative to positive, corresponding to the typical transformation from green to yellow or orange to yellow, in all varieties. Thus, the external colorations of the mutant fruits were visually similar to those of the respective ordinary genotypes (Table 1 and Table 2). These results were in good agreement with those of other orange mutants, in which the red-fleshed phenotype is not associated with alterations in the coloration of the peel [24,25,27,38].

The analysis of the internal quality parameters (°Brix and total acidity) revealed only minor differences between the K and N fruits at the initial maturity stages (October and November) (Table 1). In general, the internal maturation progressed at a slower rate in the fruits of the Valencia group (V and R, Table 2) than in those of the Navel group, in accordance with the late harvesting of the Valencia genotypes. No significant differences in the juice content of the mature fruits were recorded between the red-fleshed and the corresponding blond oranges (data not shown). Together, these results indicate that the evolution of the internal quality parameters in the red-fleshed K and R genotypes was similar to that of the ordinary varieties. Other studies with the red-fleshed variety, Cara Cara, showed lower ºBrix and maturity indices than the ordinary Navel variety [29,30], but whether these discrepancies may be related to differences in the growing or environmental conditions or to the genotype remains to be ascertained [48]. Our results indicate that under the conditions of our study, the reddish inner coloration is the main distinctive trait between the K and V fruits and the corresponding traditional varieties, whereas the external appearance, size and maturity are similar.

### 3.2. Changes in Sugars and Organic Acids in the Red-Fleshed Kirkwood Navel and Ruby Valencia and in the Ordinary Navel and Valencia Oranges

**Figure 2:**
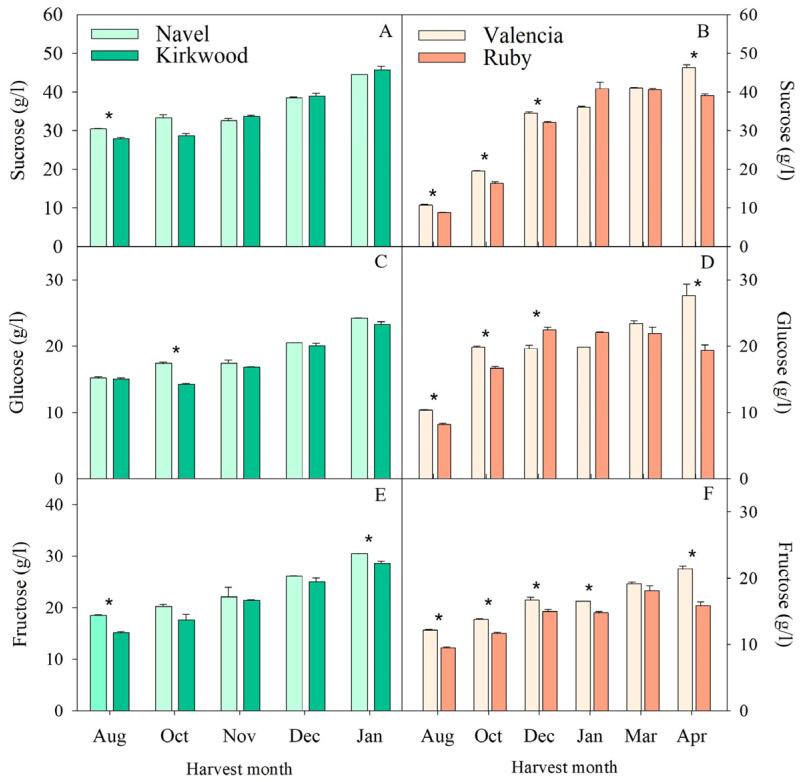
Concentrations of sucrose (**A**,**B**), glucose (**C**,**D**) and fructose (**E**,**F**) in the pulps of Navel and Kirkwood (**left panel**) and Valencia and Ruby (**right panel**) oranges during late fruit development and maturation. Results are the mean of two biological replicates ± SD. Asterisks indicate significant differences between the red-fleshed K and R and the respective ordinary oranges, N and V, for each harvest month by *t*-test (*p* ≤ 0.05).

The analysis of the main sugars in the pulps of the four genotypes revealed a progressive accumulation during development and maturation (Figure 2). The concentrations of sucrose, fructose and glucose in the Navel oranges ranged from 28–46 g/L 14–25 g/L and 17–30 g/L, respectively, whereas in the Valencia group, they were 8–46 g/L, 8–27 g/L and 9–21 g/L. The concentrations of sugars detected in these varieties were in accordance with previous reports for fruits of the Navel and Valencia varieties [35,48,49,50]. It is well known that fruit sweetness depends not only on the content of individual sugars, but also on the ratio between the main sugars [49]. In the present study, sucrose was the dominant sugar in the fruits of the four orange varieties and the ratio of sucrose, fructose and glucose was approximately 2:1:1, consistent with previous results in other orange varieties [1,49,50]. No significant differences in the sugar content were observed between the N and K fruits in most of the stages of development analyzed (Figure 2). By contrast, the V fruits contained slightly larger concentrations of sugars compared to the R fruits in August, October and December. More importantly, mature V fruits (April) accumulated 15% more sucrose and 30% more glucose and fructose than those of R (Figure 2E,F). Kafkas et al. [35] and Brasili et al. [29] also found that mature Navel oranges had higher levels of glucose and fructose, but lower levels of sucrose compared to the red-fleshed variety, Cara Cara.

**Figure 3:**
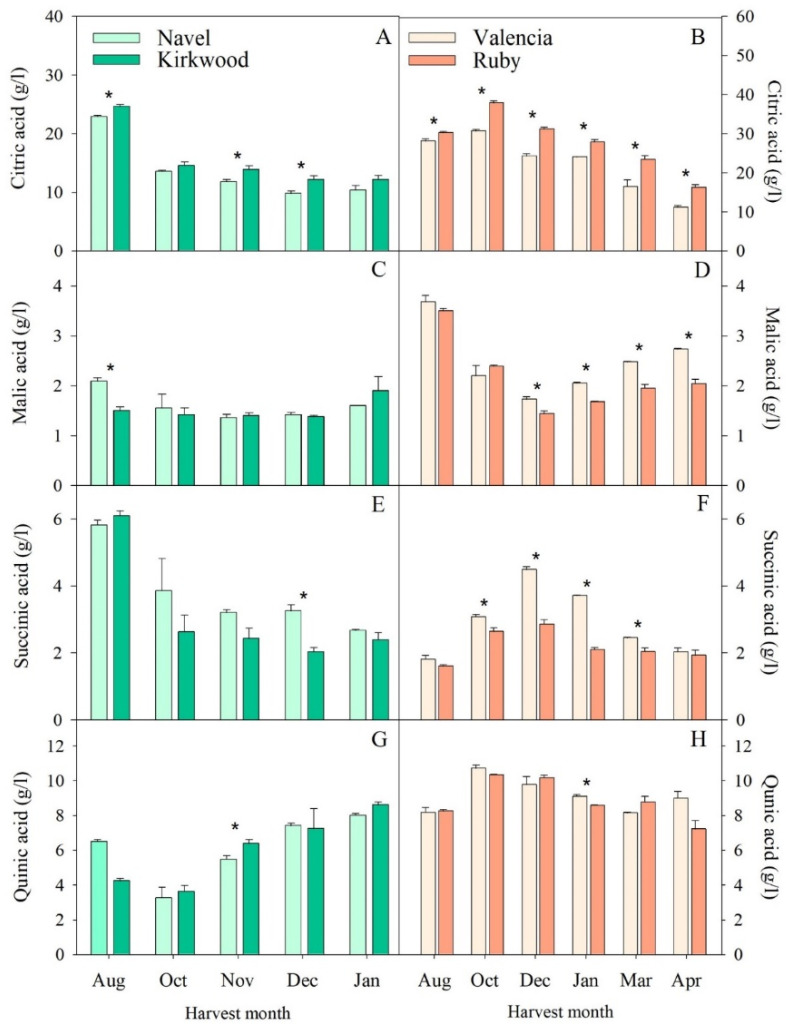
Concentrations of citric acid (**A**,**B**), malic acid (**C**,**D**), succinic acid (**E**,**F**) and quinic acid (**G**,**H**) in the pulps of Navel and Kirkwood (**left panel**) and Valencia and Ruby (**right panel**) oranges during late fruit development and maturation. Results are the mean of two biological replicates ± SD. Asterisks indicate significant differences between the red-fleshed K and R and the respective ordinary oranges, N and V, for each harvest month by *t*-test (*p* ≤ 0.05).

In this study, we detected four major organic acids in the pulps of the four varieties evaluated (Figure 3). Citric acid was the most abundant organic acid, followed by quinic, succinic and malic acid. The concentrations of the citric and succinic acid decreased in all the varieties during maturation. The malic acid remained relatively stable during maturation, whereas the quinic acid increased progressively during maturation in the N and K and remained with minor fluctuations in the V and R (Figure 3G,H). The concentrations of malic acid in the V and R fruits decreased from August to December and then experienced a gradual increase until full maturity (Figure 3D). The pattern of organic-acid accumulation in this study was relatively similar to that generally observed in fruits of other varieties [48,50].

In the orange fruits, the free acids increased rapidly in stage II of the fruits’ development and then declined in concentration due to the dilution caused by the increase in size and water content of the pulp [47], according to our results. In the two red-fleshed mutants, the accumulation of organic acids appears to have been differentially affected, whereas in the K, only occasional differences were detected and in the mature fruits, the final acid concentration was similar to that of the N oranges (Figure 3A). The concentration of citric acid in the R was between 20 and 30% higher than in the ordinary V during the whole development and maturation period (Figure 3B). In this mutant, by contrast, the malate and succinate were lower in several stages of maturation (Figure 3E,F). Nonetheless, these alterations may not have exerted a critical influence on the final taste and flavor or on the internal maturity index, probably due to the equilibrium with the other metabolites contributing to this property [48]. The accumulation of sugars and organic acids during development and maturation has been studied in detail in fruits of the red-fleshed orange, Hong Anliu [24,36]. This mutant displayed a genuine profile of sugars and organic acids, with a higher accumulation of sugars and a reduction of around fourfold in citric acid compared to the Anliu orange [36].

A comparative gene-expression analysis indicated the downregulation of the transcription of the genes relevant to citric-acid accumulation, those related to electron transport to the TCA cycle or to the proton pump involved in the transport of citrate to the vacuole [51,52]. The fact that the two red-fleshed mutants analyzed in this work did not have as severe a reduction in their citrate contents as the Hong Anliu mutant indicates that changes in these compounds may not necessarily be linked to the abnormal pigmentation of the mutants but, rather, that they are more likely the result of other pleiotropic effects on this mutant.

### 3.3. Accumulation of Ascorbic Acid, Tocopherols, Phenolic and Flavonoids Compounds in the Red-Fleshed Kirkwood Navel and Ruby Valencia and the Ordinary Navel and Valencia Oranges

**Figure 4:**
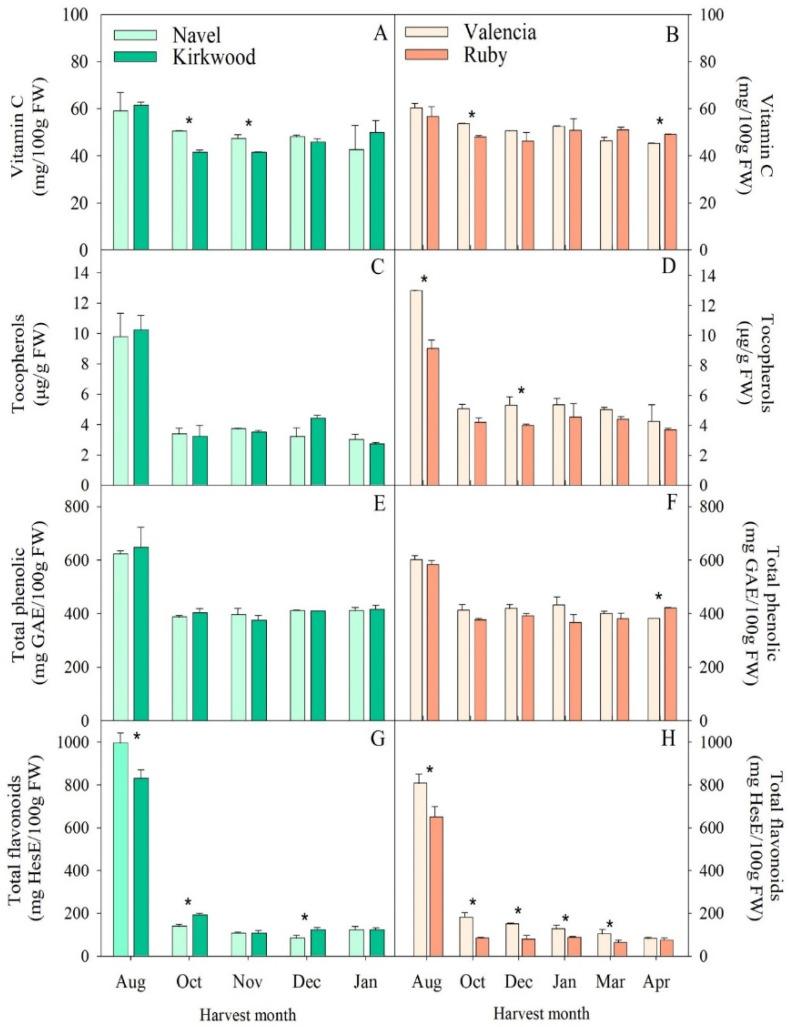
Evolution of vitamin C (**A**,**B**), tocopherols (**C**,**D**), total phenolic (**E**,**F**) and total flavonoid (**G**,**H**) contents in the pulps of Navel and Kirkwood (left panel) and Valencia and Ruby (right panel) oranges during late fruit development and maturation. Results are the mean of two biological replicates ± SD. Asterisks indicate significant differences between the red-fleshed K and R and the respective ordinary oranges, N and V, for each harvest month by *t*-test (*p* ≤ 0.05).

The concentrations of ascorbic acid and tocopherols, as well as those of phenolics and flavonoids, in the pulps of the red-fleshed oranges, K and R, and the respective blond genotype, were also analyzed during development and maturation. The orange fruit is recognized as a significant source of vitamin C. The concentrations of ascorbic acid in the pulps of Navel and Valencia oranges were relatively similar and, in general, declined moderately during maturation (Figure 4A,B). These results are consistent with those found in other citrus varieties, in which the changes in this vitamin were mainly variety-dependent and related to the maturation stage of the fruit [14,41]. However, other studies indicated that Cara Cara fruits contained lower vitamin C content than ordinary Navel oranges [30,35], pointing out that differences in the content of this vitamin in the red-fleshed mutants appear to be genotype-dependant but are not associated with its distinctive coloration.

Information about the tocopherol content in citrus fruits is very limited, and the regulation of tocopherols accumulation in fruits of different citrus species have only been investigated in recent studies [16,53,54]. The α-Tocopherol is the predominant form of tocopherol (around 99% of the total) in the pulps of the four varieties at all harvest stages, and trace amounts of γ-tocopherol were detected only in some samples (data not shown). In the current study, we found the maximum tocopherol concentration in the pulps of the immature fruits in August (10–13 µg/g FW); the values were similar in the four varieties analyzed. After this period, the total tocopherol experienced a sharp decline (54–69%) in October, after which it remained stable during the whole maturation process and without relevant differences between the blond and the red-fleshed oranges (Figure 4C,D). To our knowledge, this is the first study reporting the accumulation of tocopherols in red-fleshed orange varieties, which is in accordance with the pattern of changes reported in the pulps of other citrus species [54].

The analysis of the total phenolics and flavonoids in the pulps of the four orange varieties revealed that the immature fruits (August) contained the highest concentrations, which that thereafter (Figure 4). Previous studies have also reported a decrease in flavonoid concentrations during fruit development and the maturation of orange fruits [11,28]. These changes have been related to the oxidation of polyphenols by the polyphenol oxidase during maturation [55]. Brasili et al. [29] and De Ancos et al. [30], using the Folin–Ciocalteu assay, found similar total phenolic contents in the juices of ordinary Navel and Cara oranges. Nonetheless, other studies reported higher phenolic levels in the pulp of Cara than in that of ordinary Navel [30]. These discrepancies indicate that the growing and environmental conditions of the samples are important sources of the variability of these bioactive compounds [48].

Although the pattern of flavonoid accumulation during maturation was similar to that of the total phenolics, it is interesting that the immature fruits of the two red-fleshed oranges had lower flavonoid levels than those of the ordinary oranges (Figure 4G,H). Moreover, the concentration of these compounds in R remained below that of the ordinary V over most of the maturation period. These results are consistent with those obtained by Chen et al. [28], in which the contents of hesperidin and narirutin, the major flavonoids in orange fruits, were higher in the blond-fleshed Seike Navel and Anliu oranges than in the red-fleshed Cara Cara and Red Anliu, respectively. Whether these differences in total flavonoids may be associated with the pigmentation of the red-fleshed varieties should be further investigated.

To ascertain the quantitative and qualitative differences in flavonoids between the ordinary and the red-fleshed oranges, we analyzed the composition of the main flavonoids in the pulps of the mature fruits harvested in January for the Navel varieties and in April for those of the Valencia (Table 3). Five flavanones (hesperidin, dydimin, narirutin, naringin and eriocitrin) and one flavonol (rutin) were identified in all the samples. The major flavonoid in all the varieties was hesperidin, followed by narirutin. These results agreed with those reported in the literature [28,30,56,57]. However, the relative amount and proportion for each flavonoid is very variable, depending on the variety, maturity state, origin and climatic conditions [7]. In this study, the flavonoid contents varied slightly between the V and R fruits. The V contained higher amounts of rutin and naringin, but lower amounts of narirutin, than the R oranges (Table 3). No significant differences were detected between the R and N oranges and, in general, the total flavonoid contents were similar between the red-fleshed and the corresponding ordinary varieties (Table 3). It should be noted that other studies reported inconsistent differences in flavonoid contents between blond oranges and the red-fleshed oranges, Hong Anliu or Cara Cara [28,30]. Thus, it is likely that differences in the growing and climatic conditions, rootstock, harvest time and other variables have a pivotal influence on the contents of these bioactives. Since the fruits used in the current study were grown in the same orchard and harvested under identical conditions, our results are more likely to reflect likely genotype differences than environment-related influences.

### 3.4. Hydrophilic and Lipophilic Antioxidant Capacity in the Red-Fleshed Kirkwood Navel and Ruby Valencia and the Ordinary Navel and Valencia Oranges

**Figure 5:**
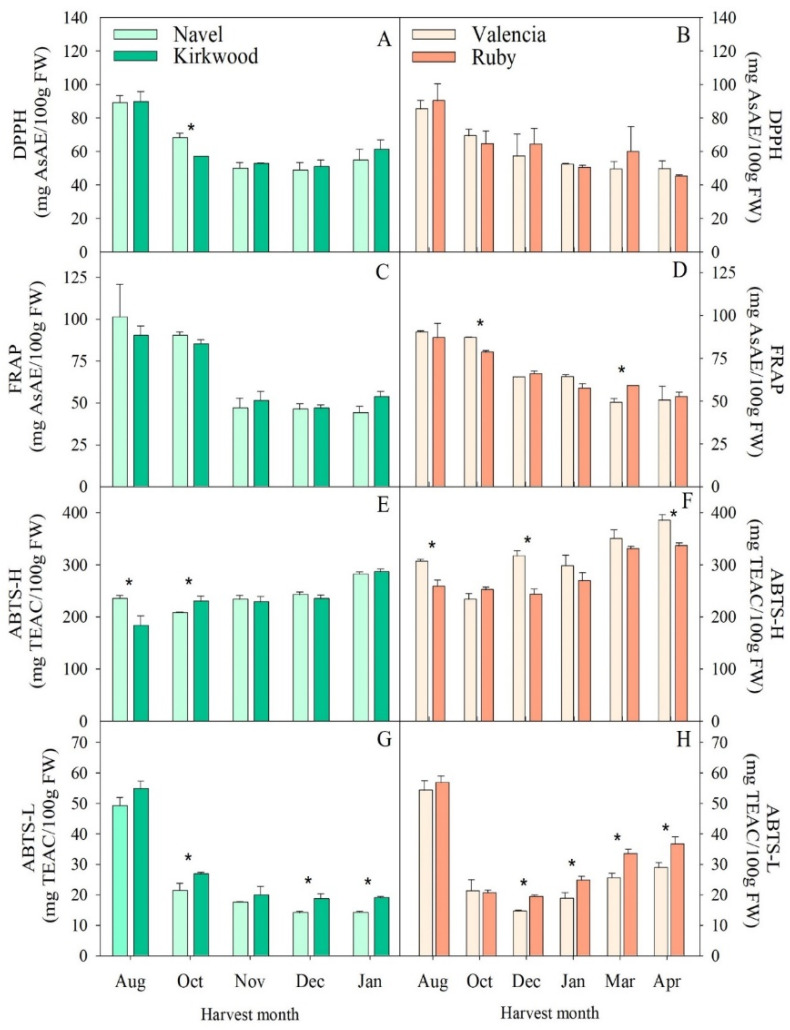
Evolution of the antioxidant capacity, evaluated by DPPH (**A**,**B**), FRAP (**C**,**D**), ABTS-H (**E**,**F**) and ABTS-L (**G**,**H**) assays, in the pulps of Navel and Kirkwood (**left panel**) and Valencia and Ruby (**right panel**) oranges during late fruit development and maturation. Results are the mean of two biological replicates ± SD. Asterisks indicate significant differences between the red-fleshed K and R and the respective ordinary oranges, N and V, for each harvest month by *t*-test (*p* ≤ 0.05).

One of the limitations in the interpretation of the antioxidant capacity of citrus fruits is the determination of the relative contribution of lipophilic compounds, such as carotenoids and tocopherols, to the antioxidant capacity, since most studies focus on the antioxidant contribution of hydrophilic compounds, such as vitamin C and flavonoids [14,58]. In this work, we assayed the evolution of the HAC and LAC in the four varieties during fruit development and maturation. The analysis of the HAC assessed by DPPH and FRAP assays showed a maximum antioxidant capacity in August, which declined progressively throughout the fruits’ growth and maturation in the four varieties (Figure 5A–D). However, the ABTS assay of the hydrophilic extracts revealed an increasing antioxidant capacity during the fruits’ maturation (Figure 5E,F). This discrepancy in the hydrophilic antioxidant capacity may indicate differences in the main compounds contributing to the activity in the different assays and in their changes during maturation. In fruits of other citrus species, variable patterns in the hydrophilic antioxidant capacity have been reported, such as a progressive increment in Yuzu (*Citrus junos*) [59], or a slight decrease in the fruits of lemon [55] and oranges [60].

Our results revealed that the contents of vitamin C positively correlated with the DPPH (r^2^ = 0.79) and FRAP (r^2^ = 0.67). Moreover, the total phenolic and flavonoid contents displayed a good positive correlation with the DPPH (r^2^ = 0.86 and r^2^ = 0.89, respectively) and FRAP activity (r^2^ = 0.65 and r^2^ = 0.72, respectively) (Table S2). These results indicate that vitamin C and polyphenols are major contributors to the HAC in all the genotypes evaluated, which is in agreement with previous observations [14,30,61]. However, the ABTS-H did not correlate significantly with either the vitamin C or the polyphenols (Table S2). This result might indicate that the scavenging capacity of the ABTS•+ of the hydrophilic fraction is likely due to individual phenolics/flavonoids rather than the total concentration [14,61]. The comparison of the HAC values by the different assays revealed that, in general, there were negligible differences between the red-fleshed and the ordinary genotypes. The V fruits presented 15–20% more HAC by ABTS than the R in the fruits harvested in August, December and April (Figure 5F). These results are consistent with the higher amount of total flavonoids detected in the pulp of the V in the several stages of development (Figure 4H). Several studies reported higher antioxidant capacity in the pulp and juice of ordinary Navel than those of Cara Cara; this was probably related to the higher levels of vitamin C [29,30,56]. In previous studies, we described higher HAC in Ruby oranges than in the blond Valencia, even though the vitamin C concentrations were similar [14]. Therefore, it is likely that the large variability in the composition of the bioactive compounds in the different orange varieties, which are strongly influenced by fruit maturity and environmental and agronomical growing conditions, may account for the discrepancies in the analysis of the antioxidant capacity among the different studies [6,56].

On the other hand, the analysis of the LAC, measured by ABTS (ABTS-L), revealed the highest values in the four varieties in immature fruits (August), which dropped sharply in October, after which they remained relatively constant or experienced a slight increase during maturation (Figure 5). Interestingly, the lipophilic ABTS activity was higher in several stages of fruit maturity in the red-fleshed fruits of K and R in comparison with the corresponding blond oranges (Figure 5G,H). As far as we know, this is the first study to investigate the LAC in the pulps of citrus fruits during the whole process of maturity.

The ABTS-L showed a significant positive Pearson’s correlation with the concentration of tocopherols (r^2^ = 0.85) (Table S2). Tocopherols are efficient ROS scavengers and, therefore, their abundance in citrus fruit can play an important role in the total antioxidant capacity of different species and varieties [54]. Our Pearson’s analysis also showed a good positive correlation coefficient with phenolics (r^2^ = 0.87) and flavonoids (r^2^ = 0.85). Several reports have obtained an efficient extraction of phenolics and flavonoids from orange tissues using ethyl acetate as a solvent [62,63]. Since, in the current study, ethyl acetate was the solvent used to extract the lipophilic compounds to test the LAC by ABTS assay, it is reasonable to assume a certain contribution of these compounds to this activity. More importantly, we recently reported that the reddish coloration of the pulps of K and R fruits is due to the accumulation of the carotene, lycopene, which is known to have significant antioxidant capacity [64,65], as well as to high amounts of the colorless carotenes, phytoene and phytofluene [38]. Since carotenoids make a significant contribution to the antioxidant capacity in the lipophilic fractions [58,61,66], it is likely that the higher lipophilic capacity found in the mature fruits of the red-fleshed oranges may be due to their specific carotenoid content and composition [14].

Overall, these results indicate that the pulp extracts of citrus fruits can effectively scavenge different types of free radicals under in vitro conditions. The variability of the assays performed suggests that multiple mechanisms and different bioactive compounds may contribute to their antioxidant capacity. For instance, Liu et al. [67] reported that the combination of lycopene + vitamin E + vitamin C showed the highest synergy in the DPPH assay, suggesting that the mechanisms of this mixture are associated with the transfer of electrons from the carotenoid to the α-tocopheroxyl radical to regenerate tocopherol, and from vitamin C to the resulting carotenoid radical cation to regenerate the carotenoids. Although the classical antioxidant methods used in the present study are based on different chemical mechanisms, all the citrus varieties studied in this work followed a similar trend and exhibited relatively similar values in terms of total antioxidant capacity. Therefore, the powerful antioxidant properties attributed to orange fruits are the result of the combination of phytochemicals and synergistic mechanisms [57]. Stahl et al. [22] found that mixtures of carotenoids are more effective than single compounds in preventing oxidative damage in multilamellar liposomes. Additionally, Chen et al. [68] demonstrated that combinations of carotenoids (lycopene and lutein) with flavonoids enhanced antioxidant capacity in cell cultures compared with individual components.

### 3.5. Singlet Oxygen Absorption Capacity (SOAC) and Its Relationship with Carotenoid Concentrations in the Red-Fleshed Kirkwood Navel and Ruby Valencia and the Ordinary Navel and Valencia Oranges

Singlet oxygen (^1^O_2_) is a reactive oxygen species (ROS) generated in biological systems, which reacts with many biological targets, inducing the degradation of cellular components. Carotenoids are efficient quenchers of ^1^O_2_ in biological systems [69]. The antioxidant activity of carotenoids depends on the number of conjugated double bonds present in their chemical structure. Thus, it is widely accepted that lycopene is the carotenoid with the greatest antioxidant activity, since it features the largest number of conjugated double bonds [70].

**Figure 6:**
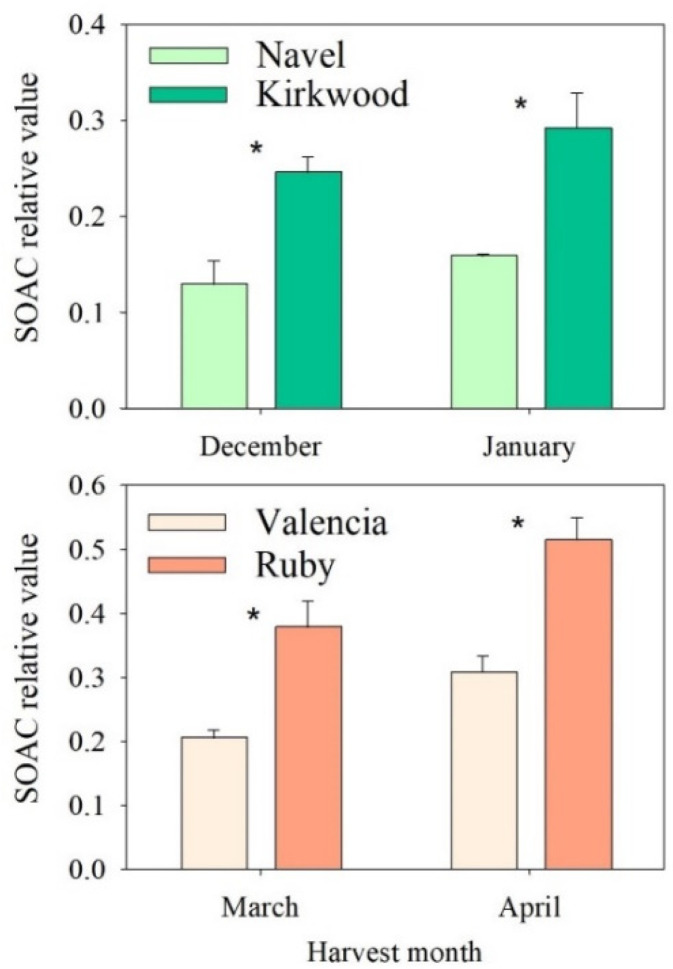
Singlet oxygen absorption capacity (SOAC) in the pulps of Navel and Kirkwood oranges (**upper panel**) harvested in December and January and Valencia and Ruby oranges (**lower panel**) harvested in March and April. Results are the mean of two biological replicates ± SD. Asterisks indicate significant differences between the red-fleshed K and R and the respective ordinary orange varieties, N and V, for each harvest month by *t*-test (*p* ≤ 0.05).

In this work, we used the SOAC assay [69] to further analyze the contribution of carotenoids to the antioxidant capacity of the red-fleshed varieties. The SOAC was determined in the pulps of mature fruits harvested in December and January for the N and K oranges and in March and April for the V and R oranges. The K and R fruits a showed 40–50% higher SOAC capacity than the N and V fruits for the two maturation stages analyzed (Figure 6).

The carotenoid contents and compositions in the pulps of these samples were analyzed to ascertain their potential relationships with the differences in SOAC capacity. The total carotenoids were 12 and 18 times higher in the mature K and V fruits than in the N and V, respectively (Tables S3 and S4). The carotenoid profiles of the ordinary blond oranges mainly composed violaxanthin as the major carotenoid, followed by lower amounts of luteoxanthin, anteraxanthin, β-cryptoxanthin, lutein and linear carotenes. The pulps of the red-fleshed oranges contained large amounts of phytoene and phytofluene, moderate concentrations of lycopene (7–9 µg/g FW) and approximately 40–50% lower amounts of violaxanthin than the ordinary varieties. In both red-fleshed oranges, low concentrations of β-carotene, with recognized pro-vitamin A activity, were detected, but not in the ordinary oranges (Tables S3 and S4). The carotenoid compositions in the R and K and the higher content of most of the carotenoids in the R than in the K were in accordance with our previous results [38].

The correlation analysis between the SOAC and the content of total carotenoids, phytoene+phytofluene and lycopene, integrating data from the four varieties and the two harvest dates, revealed a strong positive correlation with the variables (Table S5). These results indicate that the higher SOAC in the red-fleshed oranges was likely due to the difference in the content of these carotenoids. It has been proposed that lycopene is the most effective scavenger of singlet oxygen, followed by phytofluene and phytoene, by both electron transfer and free-radical deactivation [65]. Therefore, it is reasonable to assume that, together with lycopene, the unusually high levels of colorless carotenes contribute to a higher level of ^1^O_2_-quenching activity in the red-fleshed oranges.

### 3.6. Multivariate Analysis (PCA) of Bioactive Compounds, Sugars, Organic Acids and Antioxidant Capacity in the Red-Fleshed Kirkwood Navel and Ruby Valencia and the Ordinary Navel and Valencia Oranges during Fruit Development and Maturation

**Figure 7:**
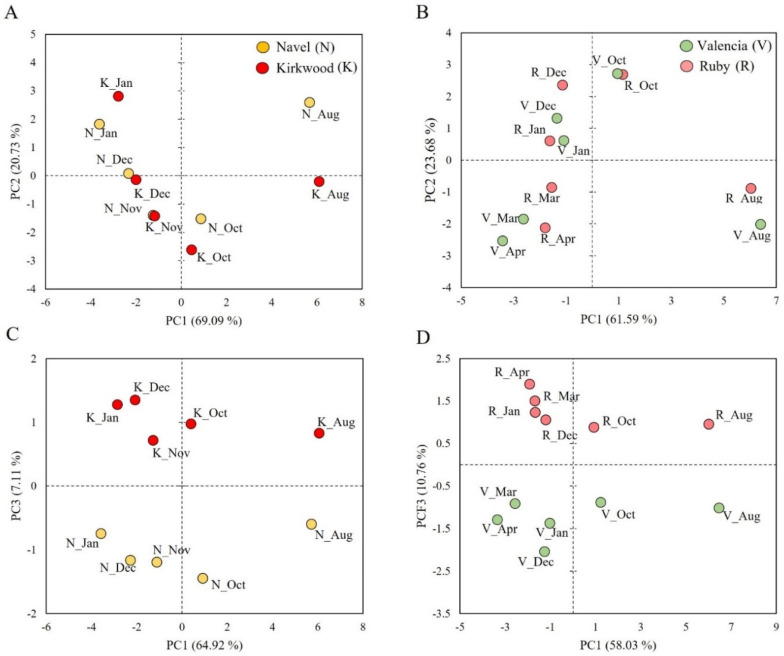
Principal component analysis (PCA) of the variables analyzed (vitamin C, tocopherols, total phenolics, total flavonoids, DPPH, FRAP, ABTS-H and ABTS-L) in Navel and Kirkwood oranges (**A**), and Valencia and Ruby oranges (**B**). Principal component analysis (PCA) of the variables analyzed and total carotenoid contents adapted from Zacarías-García et al. [38], in Navel and Kirkwood oranges (**C**), and Valencia and Ruby oranges (**D**). Aug: August; Oct: October; Nov: November; Dec: December; Jan: January; Mar: March; Apr; April.

A principal component analysis (PCA) was carried out to explore the relationship between the contents of the different compounds (sucrose, glucose, fructose, citric acid, malic acid, succinic acid, quinic acid, vitamin C, tocopherols, total phenolics and total flavonoids) and the antioxidant activity (DPPH, FRAP, ABTS-H and ABTS-L) (Figure 7A,B). In the first analysis, the two principal components (PCs) explained 89.82% and 85.27% of the total variance, for the Navel and Valencia oranges, respectively (Figure 7A,B). The samples were separated by the maturity stage of the fruits, indicating that this was the main factor in their variability.

In the second PCA analysis, including total carotenoids [38], the samples of the red-fleshed varieties were well separated from the blond oranges (Figure 7C,D). Interestingly, the PC1 explained 64.92% (Figure 7C) and 58.03% (Figure 7D) of the variance, separating the samples according to the stage of harvest, whereas the PC3 accounted for 7.11% (Figure 7C) and 10.76% (Figure 7D) of the variance, discriminating the samples by their carotenoid contents. Together, these results indicate that the concentrations of the different compounds analyzed and their respective antioxidant capacities may discriminate the stage of maturity of the different orange varieties (both red and blond oranges), but the discrimination between the red and the blond oranges was achieved through the carotenoid content.

## 4. Conclusions

The analysis of the quality attributes, bioactive compounds and antioxidant activities from the last stages of fruit development to full maturity revealed that the new red-fleshed varieties of sweet orange, Kirkwood Navel and Ruby Valencia, displayed, under Mediterranean growing conditions, a pattern and timing of maturation and a harvesting date similar to those of the ordinary Navel and Valencia varieties. No relevant differences were detected in the accumulation of sugars, organic acids, phenolics, flavonoids, vitamin C or tocopherols between the red Kirkwood and the blond Navel. However, the Ruby Valencia fruits had lower levels of sugars, malic acid and succinic acid and higher levels of citric acid than the ordinary Valencia fruits. The main feature of both red-fleshed oranges was the presence of higher lipophilic antioxidant capacity (LAC) and singlet oxygen absorption capacity (SOAC) than in the ordinary oranges, which was probably due to their lycopene content and, in particular, to their high levels of phytoene and phytofluene. Taken together, the results suggest that these novel red-fleshed orange varieties offer new products to the fresh-citrus market and juice-processing industries, with improved properties primarily derived from their carotenoid content and composition, as well as their lipophilic and singlet oxygen antioxidant capacity, without any detrimental effects on their other fruit-quality attributes. Further experiments with cell cultures, in vivo systems or clinical trials would be appropriate to evaluate the additional health-related benefits of these red-fleshed orange fruits.

## Figures and Tables

**Table 1:** Changes in fruit diameter and height, peel and pulp color and internal quality parameters of Navel and the red-fleshed Kirkwood oranges during development and maturation.

Parameter	Navel				
	Aug	Oct	Nov	Dec	Jan
Diameter (cm)	4.63 ± 0.14	7.27 ± 0.16	7.18 ± 0.14	7.39 ± 0.27	7.70 ± 0.19
Height (cm)	4.58 ± 0.15	6.81 ± 0.18	6.81 ± 0.15	7.20 ± 0.27 *	7.55 ± 0.21
Peel color (a/b)	-	−0.72 ± 0.02	0.49 ± 0.04	0.66 ± 0.04	0.76 ± 0.03
Pulp color (a/b)	-	−0.24 ± 0.01 *	−0.12 ± 0.07 *	−0.06 ± 0.01 *	0.00 ± 0.01 *
TSS (°Brix)	-	10.65 ± 0.07 *	11.55 ± 0.07	12.55 ± 0.21	12.75 ± 12.8
TA (mg CA/100 mL)	-	1.28 ± 0.01 *	1.01 ± 0.04	0.86 ± 0.05	0.84 ± 0.02
MI (TSS/TA)	-	8.32 ± 0.06 *	11.47 ± 0.52 *	14.59 ± 0.03	15.11 ± 0.44
Parameter	Kirkwood				
	Aug	Oct	Nov	Dec	Jan
Diameter (cm)	4.76 ± 0.14	7.00 ± 0.24	6.84 ± 0.11	7.11 ± 0.14	7.54 ± 0.18
Height (cm)	4.66 ± 0.19	6.43 ± 0.12	6.49 ± 0.11	6.53 ± 0.18	7.16 ± 0.11
Peel color (a/b)	-	−0.72 ± 0.02	0.37 ± 0.02	0.57 ± 0.03	0.77 ± 0.02
Pulp color (a/b)	-	0.66 ± 0.07	0.41 ± 0.05	0.46 ± 0.05	0.54 ± 0.03
TSS (°Brix)	-	10.15 ± 0.07	11.45 ± 0.35	12.55 ± 0.11	12.80 ± 0.01
TA (mg CA/100 mL)	-	1.46 ± 0.01	1.14 ± 0.02	0.87 ± 0.03	0.83 ± 0.04
MI (TSS/TA)	-	6.95 ± 0.01	9.98 ± 0.21	14.42 ± 0.10	15.29 ± 0.16

Asterisks indicate significant differences between the red-fleshed orange K and the N orange for the same harvest month (*p* ≤ 0.05, *t*-test). TSS: total soluble solids; TA: titratable acidity; CA: citric acid; MI: maturity index.

**Table 2:** Changes in fruit diameter and height, peel and pulp color, and internal-quality parameters of Valencia and the red-fleshed Ruby oranges during development and maturation.

Parameters	Valencia					
	Aug	Oct	Dec	Jan	Mar	Apr
Diameter (cm)	4.28 ± 0.09	6.18 ± 0.09 *	6.44 ± 0.08 *	6.59 ± 0.17	6.91 ± 0.11	6.95 ± 0.13
Height (cm)	4.24 ± 0.10 *	6.10 ± 0.09	6.25 ± 0.12	6.46 ± 0.11	6.81 ± 0.09	7.08 ± 0.14
Peel color (a/b)	-	−0.75 ± 0.01	0.39 ± 0.03 *	0.66 ± 0.03	0.46 ± 0.02	0.66 ± 0.04
Pulp color (a/b)	-	−0.34 ± 0.01 *	−0.16 ± 0.01 *	−0.07 ± 0.01 *	0.00 ± 0.01 *	0.06 ± 0.01 *
TSS (°Brix)	-	7.80 ± 0.02	8.66 ± 0.05	9.02 ± 0.03 *	10.70 ± 0.21	11.50 ± 0.10
TA (mg CA/100 mL)	-	1.92 ± 0.09	1.44 ± 0.17	1.24 ± 0.03	1.10 ± 0.06	0.99 ± 0.07
MI (TSS/TA)	-	4.06 ± 0.08 *	6.01 ± 0.21	7.27 ± 0.15	9.72 ± 0.16	11.60 ± 0.87
Parameters	Ruby					
	Aug	Oct	Dec	Jan	Mar	Apr
Diameter (cm)	4.13 ± 0.11	5.73 ± 0.09	5.83 ± 0.27	6.32 ± 0.08	6.52 ± 0.09	6.51 ± 0.10
Height (cm)	4.15 ± 0.11	5.83 ± 0.25	6.03 ± 0.26	6.11 ± 0.11	6.32 ± 0.10	6.59 ± 0.11
Peel color (a/b)	-	−0.79 ± 0.01	0.16 ± 0.03	0.59 ± 0.02	0.49 ± 0.03	0.67 ± 0.02
Pulp color (a/b)	-	0.72 ± 0.06	0.55 ± 0.05	0.55 ± 0.03	0.65 ± 0.03	0.60 ± 0.03
TSS (°Brix)	-	7.85 ± 0.07	9.05 ± 0.11	9.80 ± 0.03	10.86 ± 0.05	11.95 ± 0.20
TA (mg CA/100 mL)	-	1.75 ± 0.09	1.59 ± 0.06	1.50 ± 0.08	1.30 ± 0.17	1.05 ± 0.04
MI (TSS/TA)	-	4.48 ± 0.08	5.69 ± 0.30	6.53 ± 0.34	8.35 ± 1.10	11.38 ± 0.54

Asterisks indicate significant differences between the red-fleshed orange R and the V orange for the same harvest month (*p* ≤ 0.05, *t*-test). TSS: total soluble solids; TA: titratable acidity; CA: citric acid; MI: maturity index.

**Table 3:** Contents of individual and total flavonoids (mg/100g FW) in the pulps of mature fruits, Navel and Valencia, and the corresponding red-fleshed oranges, Kirkwood and Ruby Valencia, during development and maturation. Fruits of Navel and Kirkwood were harvested in January and those of Valencia and Ruby in April.

Flavonoid	Navel	Kirkwood	Valencia	Ruby
Rutin	2.23 ± 0.17	2.69 ± 0.60	2.21 ± 0.02 *	2.04 ± 0.02
Eriocitrin	0.98 ± 0.09	1.08 ± 0.13	0.84 ± 0.12	0.76 ± 0.01
Narirutin	7.58 ± 1.05	9.08 ± 1.90	3.76 ± 0.15 *	4.45 ± 0.07
Naringin	0.28 ± 0.03	0.30 ±0.07	0.63 ± 0.05 *	0.45 ± 0.02
Hesperidin	46.05 ± 9.02	60.11 ± 4.72	53.93 ± 5.72	53.48 ± 1.35
Dydimin	0.85 ± 0.13	1.17 ± 0.26	0.46 ± 0.12	0.72 ± 0.07
Total	57.96 ± 10.43	74.43 ± 7.41	61.84 ± 6.17	61.90 ± 1.52

Results are the mean of two biological replicates ± SD. Asterisks indicate significant differences between the red-fleshed oranges, K and R and the ordinary varieties, N and V, respectively (*p* ≤ 0.05, *t*-test).

## Data Availability

All the data are contained in the article and Supplementary Materials.

## Supplementary Materials

The following supporting information can be downloaded at: https://www.mdpi.com/article/10.3390/antiox11101905/s1. Table S1: Spectroscopic characteristics of the main carotenoids identified in the chromatograms of the pulp extracts of Navel, Kirkwood, Valencia and Ruby orange varieties; Table S2: Pearson’s correlation coefficients (r^2^) among vitamin C, tocopherols, total phenolics, total flavonoids, DPPH, FRAP, ABTS-H and ABT-L; Table S3: Carotenoid contents and compositions (µg/g) in the pulps of Navel and Kirkwood fruits harvested in December and January. ND: non-detected; Table S4: Carotenoid contents and compositions (µg/g) in the pulps of Valencia late and Ruby fruits harvested in March and April. ND: non-detected; Table S5: Pearson’s correlation coefficients (r^2^) among total carotenoids (TC), phytoene + phytofluene content (PE+PF), lycopene (LYC) and singlet oxygen absorption capacity (SOAC) evaluated in the pulps of Navel and Kirkwood fruits harvested in December and January and in the pulps of Valencia and Ruby fruits harvested in March and April.
