# Supplementary material for: Bioactive Compounds, Nutritional Quality and Antioxidant Capacity of the Red-Fleshed Kirkwood Navel and Ruby Valencia Oranges
Source: Antioxidants (Basel). 2022 Sep 26;11(10):1905. doi: 10.3390/antiox11101905 (PMC9598057; doi:10.3390/antiox11101905)
Supplement: Supplementary file 1 [file antioxidants-11-01905-s001.zip › antioxidants-1908907-supplementary-final/Table Supplementary S1.pdf]

Table S1. Spectroscopic characteristics of the main carotenoids identified in the chromatograms of the pulp extracts of Navel, Kirkwood, Valencia and Ruby orange varieties.

**Carotenoid**	**UV-Vis absorption maxima (nm)**
*Phytoene	285
*Phytofluene	331, 346, 364
ζ-carotene	296(*Z*), 379, 400, 425
Neurosporene	s, 433, 461
* Lycopene	446, 472, 504
δ-carotene	431, 457, 487
*Lutein	s, 444, 472
*β-carotene	s, 452, 478
*β-cryptoxanthin	423, 450, 479
*Zeaxanthin	s, 450, 475
*Antheraxanthin	s, 441, 469
*All-*E*-violaxanthin	415, 438, 468
*9-*Z*-violaxanthin	328(*Z*), 412, 436, 464
Luteoxanthin	396, 421, 448
Mutatoxanthin	s, 420, 445

*Identified using authentic standards; s, shoulder.
