# Supplementary material for: Bioactive Compounds, Nutritional Quality and Antioxidant Capacity of the Red-Fleshed Kirkwood Navel and Ruby Valencia Oranges
Source: Antioxidants (Basel). 2022 Sep 26;11(10):1905. doi: 10.3390/antiox11101905 (PMC9598057; doi:10.3390/antiox11101905)
Supplement: Supplementary file 1 [file antioxidants-11-01905-s001.zip › antioxidants-1908907-supplementary-final/Table Supplementary S2.pdf]

Table S2. Pearson's correlation coefficients (r^2) among vitamin C, tocopherols, total phenolics, total flavonoids, DPPH, FRAP, ABTS-H and ABT-L.

	VIT C	TOC	TP	TF	DPPH	FRAP	ABTS-H	ABTS-L
VIT C	-	0.85*	0.82*	0.80*	0.79*	0.67*	-0.14	0.72*
TOC	0.85*	-	0.91*	0.92*	0.82*	0.65*	-0.07	0.85*
TP	0.82*	0.91*	-	0.97*	0.86*	0.65*	-0.24	0.87*
TF	0.80*	0.91*	0.97*	-	0.88*	0.72*	-0.28	0.86*
DPPH	0.79*	0.82*	0.86*	0.89*	-	0.85*	-0.39	0.79*
FRAP	0.67*	0.65*	0.65*	0.72*	0.85*	-	-0.43*	0.63*
ABTS-H	-0.14	-0.07	-0.24	-0.28	-0.39	-0.43*	-	-0.02
ABTS-L	0.72*	0.85*	0.87*	0.85*	0.79*	0.63*	-0.02	-

Asterisk indicates significant Pearson's correlation coefficient at level $p \leq 0.05$.
