# Supplementary material for: Bioactive Compounds, Nutritional Quality and Antioxidant Capacity of the Red-Fleshed Kirkwood Navel and Ruby Valencia Oranges
Source: Antioxidants (Basel). 2022 Sep 26;11(10):1905. doi: 10.3390/antiox11101905 (PMC9598057; doi:10.3390/antiox11101905)
Supplement: Supplementary file 1 [file antioxidants-11-01905-s001.zip › antioxidants-1908907-supplementary-final/Table Supplementary S3.pdf]

Table S3. Carotenoid content and composition (µg/g FW) in the pulp of Navel and Kirkwood fruits harvested in December and January. ND: non detected.

	Navel		Kirkwood	
Carotenoids	December	January	December	January
Phytoene	0.13 ± 0.04	0.35 ± 0.04	45.71 ± 0.60	56.91 ± 0.76
Phytofluene	0.03 ± 0.01	0.07 ± 0.01	9.27 ± 0.48	11.15 ± 1.58
ζ-carotene	0.02 ± 0.01	0.03 ± 0.02	0.20 ± 0.08	0.15 ± 0.01
Neurosporene	ND	ND	0.31 ± 0.06	0.38 ± 0.16
Lycopene	ND	ND	6.92 ± 0.18	8.83 ± 0.53
δ-carotene	ND	ND	0.08 ± 0.03	0.10 ± 0.01
Lutein	0.22 ± 0.04	0.33 ± 0.05	0.20 ± 0.10	0.27 ± 0.01
β-carotene	ND	ND	0.20 ± 0.02	0.47 ± 0.02
β-cryptoxanthin	0.23 ± 0.05	0.54 ± 0.26	0.31 ± 0.06	0.34 ± 0.03
Zeaxanthin	0.09 ± 0.02	0.25 ± 0.08	0.17 ± 0.06	0.44 ± 0.01
Anteraxanthin	0.47 ± 0.02	0.87 ± 0.34	0.12 ± 0.01	0.64 ± 0.01
Violaxanthin	3.70 ± 0.18	3.55 ± 1.34	1.85 ± 0.06	2.05 ± 0.20
Luteoxanthin	0.56 ± 0.16	0.92 ± 0.19	0.17 ± 0.03	0.21 ± 0.03
Mutatoxanthin	0.03 ± 0.01	0.14 ± 0.02	ND	ND
Total carotenoids	5.49 ± 0.20	7.10 ± 1.96	65.51 ± 0.96	81.94 ± 0.86
