# Supplementary material for: Bioactive Compounds, Nutritional Quality and Antioxidant Capacity of the Red-Fleshed Kirkwood Navel and Ruby Valencia Oranges
Source: Antioxidants (Basel). 2022 Sep 26;11(10):1905. doi: 10.3390/antiox11101905 (PMC9598057; doi:10.3390/antiox11101905)
Supplement: Supplementary file 1 [file antioxidants-11-01905-s001.zip › antioxidants-1908907-supplementary-final/Table Supplementary S4.pdf]

Table S4. Carotenoid content and composition (µg/g FW) in the pulp of Valencia and Ruby fruits harvested in March and April. ND: non detected.

	Valencia		Ruby	
Carotenoids	March	April	March	April
Phytoene	0.57 ± 0.01	1.05 ± 0.04	103.66 ± 6.27	133.12 ± 1.08
Phytofluene	0.15 ± 0.01	0.18 ± 0.01	17.82 ± 0.15	18.42 ± 3.60
ζ-carotene	0.17 ± 0.02	0.29 ± 0.02	0.40 ± 0.17	0.62 ± 0.13
Neurosporene	ND	ND	0.56 ± 0.18	0.33 ± 0.02
Lycopene	ND	ND	8.15 ± 1.30	8.58 ± 0.14
δ-carotene	ND	ND	0.17 ± 0.10	0.13 ± 0.01
Lutein	0.57 ± 0.09	0.61 ± 0.08	1.21 ± 0.09	0.32 ± 0.05
β-carotene	traces	traces	0.62 ± 0.12	0.41 ± 0.03
β-cryptoxanthin	0.55 ± 0.06	0.58 ± 0.16	0.32 ± 0.11	0.30 ± 0.10
Zeaxanthin	0.51 ± 0.05	0.66 ± 0.15	0.63 ± 0.13	0.61 ± 0.02
Anteraxanthin	1.00 ± 0.13	1.20 ± 0.12	0.89 ± 0.38	0.98 ± 0.26
Violaxanthin	2.53 ± 0.79	3.70 ± 0.11	1.54 ± 0.98	2.30 ± 0.03
Luteoxanthin	1.02 ± 0.20	0.58 ± 0.17	0.23 ± 0.04	0.23 ± 0.03
Mutatoxanthin	0.11 ± 0.07	0.18 ± 0.13	0.07 ± 0.03	0.10 ± 0.01
Total carotenoids	8.18 ± 0.84	9.12 ± 0.92	135.33 ± 5.05	166.49 ± 2.81
