# Supplementary material for: Bioactive Compounds, Nutritional Quality and Antioxidant Capacity of the Red-Fleshed Kirkwood Navel and Ruby Valencia Oranges
Source: Antioxidants (Basel). 2022 Sep 26;11(10):1905. doi: 10.3390/antiox11101905 (PMC9598057; doi:10.3390/antiox11101905)
Supplement: Supplementary file 1 [file antioxidants-11-01905-s001.zip › antioxidants-1908907-supplementary-final/Table Supplementary S5.pdf]

Table S5. Pearson's correlation coefficients (r^2) among total carotenoids (TC), phytoene + phytofluene content (PE+PF), lycopene (LYC) and singlet oxygen absorption capacity (SOAC) evaluated in the pulp of Navel and Kirkwood fruits harvested in December and January and, in the pulp of Valencia and Ruby fruits harvested in March and April.

	TC	PH+PF	LYC	SOAC
TC	-	1.00	0.90*	0.88*
PH+PF	1.00	-	0.89*	0.88*
LYC	0.90*	0.89*	-	0.71*
SOAC	0.88*	0.88*	0.71*	-

Asterisk indicates significant Pearson's correlation coefficient at level $p \leq 0.05$.
